# Notes and News

**Published:** 1888-07-28

**Authors:** 


### Notes and News.

Collected and Communicated.

It is stated that the Street Collection of the Hospital Saturday Fund is in advance of any former collection by several hundred pounds.

A meeting of the directors of the Ladies' Dwellings Company was held at the Grosvenor Hall, Buckingham Palace Road, on Wednesday afternoon.

People, even in London, still continue to seek for escaped gas with naked lights. On Sunday Mr. W. Royal, of Cologne Road, Battersea, repeated this strange folly, and was severely burnt in the face and hands in consequence.

It is said that in spite of the low temperature and the frequent rains of the last two months girls innumerable have persisted in wearing white muslin and thin cambric gowns. The doctors, it is probable, will not have suffered in consequence, whatever the girls may have done.

The election of an honorary medical officer to the Worthing Infirmary in place of Mr. J. E. Grinfield-Coxwell resigned, took place last week. The candidates were Mr. Charles E. Smith, M.D., St. Andrews, M.R.C.S. London, and Mr. William Huyton Gostling, M.D. London, M.R.C.S. London. It was sought to declare Dr. Smith ineligible on the ground of insufficient qualification. This objection was overruled by the chairman, and Dr. Smith was declared duly elected by a majority of 69 votes.

The French Chamber has before it a Bill for the regulation of the labour hours of women and childrem employed in factories. The Chamber rejected a proposal to forbid women to work; but it accepted one to require a day in seven as a day of rest both for women and children.

The sum of £5 2*s.* 8*d.* has been sent to the Mansion House for the Hospital Sunday Fund from St. Gabriel's, Canning Town, E. In a district like Canning Town, where all the inhabitants are poor, such a contribution may tell of more self-denial and true interest in hospitals than far larger sums in more prosperous districts.

An open-air dramatic performance was given last Saturday afternoon in the grounds of Sir Sydney Waterlow, at Highgate Hill, on behalf of the building fund of the Great Northern Central Hospital. The weather was not very favourable, but there was a good audience, which seemed much pleased with the performance.

May the rain have rained itself out before Wednesday, August 1st, on the afternoon of which day an open-air performance of the forest scenes from *As You Like It* is to be given at Park Lodge, Park Place Villas, Maida Hill, W., in aid of St. Mary's Hospital, Paddington. If the weather is fine, few pleasanter ways of spending an afternoon could be invented; and it is to be hoped that many people will have faith and courage enough to respond to the invitation on the bill, and make "early application" for tickets to Mr. Thomas Ryan, the secretary of St. Mary's Hospital.

Special attention has, for some time past, been given at St. Bartholomew's Hospital to that very important factor in the treatment of injured persons, the proper carriage on stretchers, etc. Recently, under the careful supervision of Surgeon Reece, Volunteer Medical Staff Corps, and Mr. Cross, the able and energetic clerk, the whole of the porters and box carriers (whose duty it is to transport the sick) have been trained by Sergeant Bond, medical staff, in the proper methods of lifting and carrying. The course extended to twenty-four lectures, and the results as shown in actual practice have been most satisfactory. It is proposed that every six months Sergeant Bond will inspect the staff, in order that the knowledge acquired may not be allowed to rust. This is quite a new departure, and certainly well worthy of attention and imitation by all our hospitals.

THE following vacancies are announced this week, the amount of salary in each case being given after the name of the institution :—

**Resident Medical Officers.**

Birmingham General Hospital. Assistant House Surgeon.—Board; no salary. Surgical qualification required.
London Temperance Hospital, Hampstead Road. Senior House Surgeon, doubly qualified.—Fifty guineas per annum, with board, etc.
Morpeth Dispensary. House Surgeon, doubly qualified.—£120 per annum, with furnished house, coals, and gas.
Queen's Hospital, Birmingham. Resident Physician.—£50 per annum, with board, etc.
Staffordshire General Infirmary. Assistant to the House Surgeon.—Board; no salary.
Sussex County Hospital. Assistant House Surgeon, doubly qualified.—Board; no salary.

**Non-resident Medical Officers.**

City of London Hospital for Diseases of the Chest, Victoria Park, E.—Assistant Physician. Candidates must be M. or F.R.C P., Lond.
Ital'an Hospital, Queen Square, Bloomsbury. Assistant Medical Officer. Candidates must be fully qualified, and able to speak Italian.
Kilkenny Union. Medical Officer, Gowran Dispensary. -£120 per annum, and fees.
Norfolk and Norwich Hospital. Assistant Surgeon.
Poplar Hospital for Accidents. Honorary Surgeon for Out-patients. Candidates must be doubly qualified.

**Secretary.**

Norfolk and Norwich Hospital. Resident Secretary and House Steward.—£100 per annum, with board, and furnished apartments.

---

MRS. ELLIS, formerly lady superintendent of the Evelina Hospital, and lady-in-charge of Miss Louisa Twining's Home for Epileptics, has instituted a home at St. Leonards for ladies suffering from nervous diseases. The home is pleasantly situated on the West Hill, and has a direct out-look over the sea, and as St. Leonards is one of the most agreeable of our southern watering-places, it will probably attract many invalids, especially as Mrs. Ellis has now resolved to receive medical and surgical as well as nervous cases. The charges are moderate, but vary according to the care and attention required.

---

THE Peterborough Dispensary and Infirmary is a prosperous as well as a useful institution. It is able not only to pay the debts incurred in the treatment of 4,078 in and out-patients, but has shown the committee's appreciation of the work done by the medical officers and matron by substantial gifts. Dr. Walker, the surgeon to the hospital, has been presented with £50, Miss Sandon (the matron) with £10, and Dr. Hubbersty (the house surgeon) with £5, the last named on his retirement from that post after holding it for three years. The appreciation of faithful service gladly rendered which is implied in these gifts is probably more valued by the recipients than the money itself.

---

MISS BAXTER, the lady superintendent of the Cork Hospital for Women and Children, must be flattered at the testimony to her worth contained in the recently-published annual report of that institution, in which it is stated that the Board of Management "desire to acknowledge the great energy and ability she has shown in carrying out the work of the institution, and at the same time to record their belief that it is mainly due to her individual exertions that the cost of working the hospital has been so exceptionally moderate." Strict economy in management seems to have been necessary, for at the end of 1886 the hospital was so deeply in debt that there was some question of closing one of the wards. This misfortune has, however, been averted, thanks to careful management, and the assistance of good friends, which the hospital seems to be rich in. One of these, Mr. W. H. Crawford, of Lakelands, had offered to the trustees a piece of land in the neighbourhood of Queen's College, which should prove a valuable gift.

THE patients' bill of fare at the London Hospital for one day is as follows: 201 lbs. of mutton, 29 chops, 17 steaks, 172 pints of beef tea, 282 lbs. of potatoes, 119 portions of greens, 351 puddings, 1,368 pints of milk, and 767 eggs. In addition to the patients there are some 300 servants and nurses. The weekly washing bill includes 4,000 sheets, 3,000 blue-checked upper sheets, 400 counterpanes, and 400 blankets. Twenty-three women and a laundryman are employed daily in the laundry. The medical stores included last year 116 tons of ice, five tons of linseed meal, and six miles of plaster.

---

"THE proposal to give lady visitors a status at the Hospital," says a Welsh contemporary, "is a two-sided question. While, on the one hand, it is desirable that the sympathy and help and delicate attention of charitably-disposed ladies should be enlisted on behalf of the patients in the hospital, on the other hand, every care should be taken to avoid officious and intrusive interference on the part of ladies in the conduct of daily life in the sick wards. It is not every charitably-disposed lady who is by nature or by education fitted to deal wisely and well with sick persons. A kindly pressure of the hand, the soft tones of a soothing voice, the display of Christian interest are admirable; they may be said to be almost curative in their effects. The opposite qualities to these, that is to say, aggresive dogmatism, or airing of superiority, cannot but be a worry and an injury to the patients. The visits of the right sort of ladies cannot be too much encouraged at the hospital, but, quite as certainly visits of ladies of the wrong kind, however well-intentioned, should be strenuously discouraged.

---

THE Clayton Hospital, Wakefield, begins its new year absolutely free of debt, the friends of the institution, led by Mr. Percy Tew, the President, and Mr. John Binks, the indefatigable Honorary Secretary, having raised a sum of £1,501 6*s.* 9*d.*, to clear off the debt that burdened it. The annual subscriptions to the hospital and Hospital Sunday collections remain *in statu quo*, but the Hospital Saturday contribution shows a slight falling off, due to a decrease in receipts from concerts. Nevertheless, the institution may be looked upon as prosperous, as well as useful. At the annual meeting of subscribers, Dr. Holdsworth, J.P., proposed a vote of thanks to Mr. Binks, whose long services and unwearying efforts had done so much to forward the interests of the charity. The only change in the staff of the hospital noted in the report is the resignation in March last of Miss Annie Armstrong, who had filled the post of matron for three years. Miss C. Noel Thompson has been elected in her place.

---

AT the last quarterly meeting of the Norfolk and Norwich Hospital, Dr. Barton was appointed honorary physician, and Dr. Michael Beverley honorary surgeon to the hospital, in place of Sir Peter Eade and Mr. Crosse, who have resigned these posts, after having held them for thirty years, but have consented to accept the posts of consulting physician and surgeon respectively. Lately the hospital has been suffering from want of water, but it is hoped that a new system of pipes, which has been constructed at a cost of £28, will relieve the difficulty, which is caused by the site of the hospital being above the level to which the waterworks company can give a constant supply of water. Should the present plan fail to augment the supply, it will be necessary to construct new tanks, which would be a rather expensive proceeding. The Board of Management have received courteous replies from Sir Henry Ponsonby and Sir Dighton Probyn, thanking them, on behalf of the Queen and the Prince of Wales, for their address of condolence on the death of the Emperor Frederick of Germany, a copy of which has, by his Royal Highness's wish, been sent to the Empress Victoria.

